# Three-dimensional culture and clinical drug responses of a highly metastatic human ovarian cancer HO-8910PM cells in nanofibrous microenvironments of three hydrogel biomaterials

**DOI:** 10.1186/s12951-020-00646-x

**Published:** 2020-06-11

**Authors:** Hong Song, Guo-hui Cai, Jian Liang, Di-shu Ao, Huan Wang, Ze-hong Yang

**Affiliations:** 1 College of Basic Medicine, Zunyi Medical University, No.201 Dalian Road, Huichuan District, Zunyi, Guizhou, 563003 China; 2 School of Resources and Environment, ABA Normal University, Shuimo Town, Wenchuan County, Aba Prefecture, Sichuan, 623002 China; 3 West China School of Basic Medical Sciences and Forensic Medicine, Sichuan University, No.17 People′s South Road, Chengdu, Sichuan 610041 China

**Keywords:** Nanofiber, Hydrogel, 3D cell culture, HO-8910PM cells, Chemosensitivity, Cell growth pattern

## Abstract

**Background:**

Ovarian cancer is a highly aggressive malignant disease in gynecologic cancer. It is an urgent task to develop three-dimensional (3D) cell models in vitro and dissect the cell progression-related drug resistance mechanisms in vivo. In the present study, RADA16-I peptide has the reticulated nanofiber scaffold networks in hydrogel, which is utilized to develop robust 3D cell culture of a high metastatic human ovarian cancer HO-8910PM cell line accompanied with the counterparts of Matrigel and collagen I.

**Results:**

Consequently, HO-8910PM cells were successfully cultivated in three types of hydrogel biomaterials, such as RADA16-I hydrogel, Matrigel, and collagen I, according to 3D cell culture protocols. Designer RADA16-I peptide had well-defined nanofiber networks architecture in hydrogel, which provided nanofiber cell microenvironments analogous to Matrigel and collagen I. 3D-cultured HO-8910PM cells in RADA16-I hydrogel, Matrigel, and collagen I showed viable cell proliferation, proper cell growth, and diverse cell shapes in morphology at the desired time points. For a long 3D cell culture period, HO-8910PM cells showed distinct cell aggregate growth patterns in RADA16-I hydrogel, Matrigel, and collagen I, such as cell aggregates, cell colonies, cell clusters, cell strips, and multicellular tumor spheroids (MCTS). The cell distribution and alignment were described vigorously. Moreover, the molecular expression of integrin β1, E-cadherin and N-cadherin were quantitatively analyzed in 3D-cultured MCTS of HO-8910PM cells by immunohistochemistry and western blotting assays. The chemosensitivity assay for clinical drug responses in 3D context indicated that HO-8910PM cells in three types of hydrogels showed significantly higher chemoresistance to cisplatin and paclitaxel compared to 2D flat cell culture, including IC_50_ values and inhibition rates.

**Conclusion:**

Based on these results, RADA16-I hydrogel is a highly competent, high-profile, and proactive nanofiber scaffold to maintain viable cell proliferation and high cell vitality in 3D cell models, which may be particularly utilized to develop useful clinical drug screening platform in vitro.

## Background

Since their inception one hundred years ago, two-dimensional (2D) cell cultures produced important data in biomedical sciences, but the limitations of 2D cell culture were evident because cells were cultivated as monolayer on flat surface, lacking cell–cell and cell–matrix interactions that were present in native tissues. Moreover, 2D-cultured cells were stretched and underwent cytoskeletal rearrangements loosing normal cell polarity, which resulted in aberrant gene and protein expression [1, 2]. Conversely, three-dimensional (3D) cell cultures were to culture cells on the extracellular matrix (ECM)-like scaffolds in a spatially relevant manner, facilitating cell–cell and cell–matrix interactions, that really mimicked the native cell microenvironment in vivo, including cell–cell adhesion or junction, cell growth patterns, and spatial cell adaptability et al. In tumor biology research, cancer cells in vivo had contacts with ECM components in all directions and interacted with other cells of the same (or different) type in their vicinity. The tumor locus was a spatial and temporal 3D cell microenvironment undergoing multifaceted tissue remodeling at extracellular, intercellular, and intracellular levels [3, 4]. For this reason, 2D surface had more and more defects compared to the physiological 3D matrix known as cell scaffold. To fill in the gap between the monolayer cell culture and 3D cell microenvironment in vivo, more and more cell-culturing scaffolds had emerged to mimic the cell microenvironment in vivo. In these cell scaffolds, Matrigel and collagen I were currently most popular matrices and cell culture gold standards for a variety of cell types, since they resembled the native ECM components and allowed cell–ECM interactions in a in vivo-like condition and produced tissue-realistic cell niches in vitro. A series of reports showed that Matrigel and collagen I were widely used as surrogates of native ECM components and played a critical role in the regulation of cell growth, differentiation, phenotype presentation and apoptosis in 3D context [5–7]. It was one exciting cell technology that 3D cell models shed light on the molecular mechanisms underlying cell–cell communication or developed multi-organ microfluidic chip platform and complex 3D cell co-culture strategies [8–10], beyond 3D cell cultures, which surely spurred the substantial efforts towards the cell scaffold-based biomimetic 3D cell culture models and encouraged much cross-disciplinary work among biologists, material scientists, tissue engineers and clinical physicians.

Designer self-assembling peptides were made from natural amino acids and formed nanofibrous cell scaffolds with different stiffness or physical rigidity by altering peptide concentration and primary amino acid sequence [11, 12]. Due to the specialized molecular design, these designer peptides might undergo spontaneous molecular self-assembly to form well-defined nanofiber networks in hydrogel (10–20 nm in nanofiber diameter with pore size between 5 and 200 nm), and surround cells in a manner similar to the native ECM in vivo, thus producing a closely true 3D microenvironment for cell proliferation, migration, differentiation and various cell patterns formation [13–15]. According to ion-complementary self-assembly mechanism, designer peptides might form a variety of cell scaffold systems that mimicked the specific ECM characteristics related to cell or tissue types in vivo [16, 17]. Furthermore, designer peptides might be functionalized to guide specific cell type to grow, attach, differentiate and migrate in 3D context [17–19]. In previous studies, RADA16-I hydrogels were successfully used for different mammalian cell cultures, including primary hepatocyte [20], bone marrow mesenchymal stem cell [21], mouse embryonic stem cell [22], adult hepatocyte [23] and leukemia cell [24], etc. In regenerative medicine community, RADA16-I designer peptide and its derivatives were widely applied for cell scaffold-based 3D cell cultures, clinical cell therapy, injured tissue repair, and elusive drug delivery, such as bone damage repair [25], spinal cord injury repair [26], instant hemostasis [27], controlled drug release nanocarriers [28], et al. Compared to other hydrogel biomaterials, including Matrigel and collagen I, designer peptide hydrogels had some prominent advantages, such as the natural amino acid constituents, single composition without impurities, massive synthesis with batches, no cytotoxicity and immunogenicity of degradation products, etc. [13, 17, 29]. These synthesized peptide components did not contain animal source pollution, excluding the effects of the complex components of biological origin on cell behaviors, except of the benefits in the clinical translational possibilities. Most notably, designer peptide hydrogels were reproduced by the changes of amino acid sequences and the charged properties and might be designed to cater for the cell characteristics or tissue type, which might provide reliable biochemical composition, tunable mechanical stiffness, proper cell functionality, and robust cell adaptability in manufacture [15, 17, 30, 31]. Thus, it was obvious that designer peptide hydrogels showed great biomedical utility and multifaceted benefits in the development of novel 3D cell models for biomedical applications and translational researches. In our previous studies [32, 33], clinical drug chemosensitivity assay was explored in ovarian cancer extensively, but the potential biochemical basis for 3D cell cultures was not precisely clarified in easy metastatic human ovarian cancer line, which could be important in current translational medicine.

Ovarian cancer was the leading cause of death from gynecologic malignancies and the fourth most common cause of cancer death with the poor 5-year survival rates among women. The regular treatment was surgical debulking followed by combination chemotherapy with cisplatin and paclitaxel [34, 35]. However, the clinical drug resistance occurrence was intense research effort elusively and the major barrier to the successful long-term treatment of this disease clinically. According to an academic consensus epilogue [36, 37], one prominent reason lay in the lack of clinically predictive cell models that mimicked early stage in the tumor metastasis process of ovarian cancer disease, since traditional 2D cell cultures and xenograft models were difficult to recapitulate the TME in vivo and capture multiple cell types or biological functionality in intraperitoneal cavity. Thus, it was an urgent task to develop more realistic 3D cell models to understand how ovarian cancer cells responded to a variety of cues from the native niche. Although popular biomaterials had been extensively used to culture ovarian cancer cells, such as Matrigel, collagen I, hyaluronic acid, and alginate et al., designer peptide hydrogel was a biomimetic type of artificial nanomaterials which combined good biocompatibility of natural biomaterials and the flexibility of chemically-synthesized polymers. It was hopeful to create the cell or tissue-specific 3D cell models and achieve clinical drug response profiles for therapeutic agents.

In this study, by virtue of designer peptide hydrogel’s profits, a high metastatic human ovarian cancer HO-8910PM cell line was utilized to establish a kind of robust 3D cell cultures in three types of hydrogel biomaterial, such as RADA16-I hydrogel, Matrigel and collagen I. The cell morphology, cell proliferation, cell aggregate growth and cell adhesion protein expression were investigated comprehensively, in addition to 3D cell chemosensitivity assay towards clinical therapeutic reagents when compared with 2D cell culture, which provided the state-of-the-art cell approach to explore 3D cell models in vitro in designer peptide hydrogel.

## Materials and methods

### Materials

RADA16-I peptide was commercially synthesized by Fmoc solid phase method and purchased from BD Biosciences (catalog No.354250, Shanghai, China), which had 16 amin acid residues sequence Ac-RADARADARADARADA-CONH_2_ (RADA16-I in short). The lyophilized powder was prepared at a concentration of 10 mg % (w/v) in sterile ultrapure water (18.2 MΩ, Millipore, Bedford, USA) and sonicated 30 min and then stored at 4 °C for use. Collagen I (catalog No. 354236, rat tail) and Matrigel (catalog No. 356234, growth factors reduced from mouse EHS sarcoma) were purchased from BD Biosciences and used for 3D cell culture assay. Sucrose, 4′6-diamidino-2-phenylindole (DAPI), Trypsin–EDTA, calcein-AM, phalloidin, BrdU, anti-BrdU monoclonal antibody (catalog No. B2531) and goat anti-mouse IgG-FITC conjugates (catalog No. BA1101) were purchased from Sigma-Aldrich (St. Louis, MO, USA). RPMI-1640 and fetal bovine serum (FBS) were obtained from Gibco Life Technology Inc. (GIBCO, Invitrogen, Carlsbad, CA, USA). E-cadherin (ab76055), N-cadherin (ab18203) and integrin β_1_ (ab179471) antibodies were obtained from Abcam (Cambridge, UK). Other assay reagents were mainly obtained from Gibco/Thermo Fisher Scientific (Rockville, MD, USA). The cell images were taken with an inverted Olympus IX71 fluorescent microscope and Leica DMILLED microscope.

## 3D cell culture

HO-8910PM cell line was used in all assays, which was purchased from Shanghai Cell Library of Chinese Academy of Sciences (Shanghai, China). HO-8910PM cells were cultivated in RPMI 1640 medium with 10% FBS and 1% penicillin–streptomycin. 2D-cultured cells were maintained at a constant temperature of 37 °C with 5% CO_2_ and 95% humidity and grown at 80% confluence and passaged for use with the digestion of 0.25% Trypsin–EDTA. When subject to 3D cell culture in RADA16-I hydrogel, Matrigel, and collagen I, the cell suspension was prepared and cell number was adjusted to develop appropriate 3D cell culture assay. HO-8910PM cells were cultured in RADA16-I hydrogel described as following. The obtained HO-8910PM cells were pelleted and resuspended with 10% sucrose. The peptide solution was mixed thoroughly with the cell suspension at a ratio of 1:9 (RADA16-I: cell suspension, v/v), which balanced the final concentrations of RADA16-I and HO-8910PM cells at 1.0 mg/mL and 1 × 10^6^/mL, respectively, in addition to maintaining the proper pH value and normal cell osmotic pressure. The cell-peptide scaffold mixture with 100 μL volume was safely transferred to the center position in a 12-well cell plate. If 24-well cell plate was ready, the volume of mixture was changed to 50 μL. RPMI 1640 medium was safely dropped along with the inner wall of cell culture plate to immerse HO-8910PM cell-peptide scaffold block. After 30 min incubation at 37 °C, the ions in medium allowed peptide scaffold to self-assemble and become hydrogelation quickly. The gelled cell blocks were constructed and overlaid with the medium and cultivated in 37 °C incubator. The medium was appropriately refreshed every other day. HO-8910PM cells were cultured in Matrigel and collagen I as reported previously [38–40]. The concentrations of Matrigel and collagen I reached 1.5 mg/mL and 1.25 mg/mL, respectively. HO-8910PM cell concentrations in collagen I and Matrigel were approximately adjusted to 1 × 10^6^/mL, respectively. The gel-cell clumps were further cultured and changed the medium at desired time interval. For tumor cell isolation in RADA16-I peptide hydrogel, the gel-cell clumps were washed several times and dissociated carefully with slow and repetitive pipetting. As to Matrigel and collagen I, the trypsin solutions with additional type IV collagenase (0.3 mg/dL) and type I collagenase (0.1 mg/dL) were added to recovery the tumor cells in 3D cell culture setting.

### Transmission electron microscopy (TEM)

RADA16-I peptide, Matrigel, and collagen I were negatively stained with uranyl acetate and analyzed by TEM as previous methods described [41]. In brief, RADA16-I peptide, Matrigel and collagen I were diluted with 1 × PBS (10 mM, pH 7.4) to 0.1, 0.06 and 0.05 mg/mL, respectively. Drops of diluted scaffold suspension were deposited onto the surface of copper grid. The sample was dried in air at room temperature. A drop of ~ 2% phosphotungstic acid (PTA) was put onto the sample for 1 min approximately, and then covered by a perforated poly (vinyl formal) (formvar) film. The sample grids were subjected to visualize on a Hitachi H-600 instrument with an acceleration voltage of 120 kV. The width and length of nanofiber scaffold were determined and evaluated by senior instructor in college core facilities.

### Cell viability and cell proliferation or BrdU labeling assay

Gel-cell clumps were maintained under normal growth conditions as described above and collected at each time point (days 1, 3, 6 and 9). Cell number cultured on each scaffold was determined by the fluorometric quantification of DNA content, using DNA fluorescence assay kit according to the manufacturer’s instructions (Invitrogen, molecular probes). Briefly, gel-cell clumps were washed with ice cold PBS buffer and recovered by Na Citrate buffer (50 mM Na Citrate and 100 mM NaCl) and stored at − 80 °C until assay. For assay in different days, the gel-cell clumps were repeatedly frozen or thawed and shook until the cell clumps completely disassociated. The samples were recovered and the fluorescence was measured in a microplate reader (Vario Skan Flash, Thermo, USA) at an emission wavelength of 460 nm and an excited wavelength of 360 nm. When compared with DNA standards included in the assay kit, HO-8910PM cell viability was evaluated in the different hydrogel scaffolds. For qualitative cell proliferation observation, gel-cell clumps were incubated in PBS containing 4 μM calcein Am to stain the viable cells and 1.5 mM propidium iodide (PI) was used to stain dead cells. An inverted Olympus IX71 fluorescent microscope was used to capture the fluorescent signaling.

Generally, cell growth was observed at the desired time points. The normal phase contrast images were obtained in a Leica DMILLED microscope. For 5-bromo-2′deoxyuridine-5′monophophate (BrdU) labelling cell proliferation assay, approximate 1 × 10^6^/mL of HO-8910PM cells were seeded on each scaffold. Gel-cell clumps were collected at days 1, 3, 6 and 9. BrdU is a synthetic nucleoside analog of thymidine. The equal amount of 100 μM BrdU reagent was added into the gel-cell clumps and additionally incubated for 18 h. HO-8910PM cells in gel-cell clumps were recovered according to the method described above. After fixing the tumor cells and denaturing cellular DNA, the obtained HO-8910PM cells were incubated with goat anti-mouse IgG-FITC conjugate for 90 min at room temperature. The soluble antibody conjugates were removed in three washing cycles. BrdU-labeled nuclei were recorded with 200 cells per cell culture plate well. The labelling percentage indexes were expressed to be a ratio of BrdU-labeled cell number in the total cell nuclei. All measurements were performed in duplicates with four wells per hydrogel.

### Cell morphology in 3D culture and tumor spheroid formation

For Phalloidin/DAPI staining, HO-8910PM cells were cultured in three types of hydrogel for day 6 or day 12, and carefully rinsed with ice cold PBS buffer three times. The gel-cell clumps were fixed with 4% paraformaldehyde and washed with PBS twice. Gel-cell clumps were then permeabilized with PBS containing 0.5% Triton X-100 for 30 min and stained for 20 min with rhodamine phalloidin (Invitrogen), and then washed with PBS buffer. F-actin of HO-8910PM cells was visually observed to identify the cell–cell interaction and cytoskeleton architecture in gel-cell clump. To visualize cell nuclei, HO-8910PM cells in 3D cell culture were treated with DAPI (0.5 μg/mL) at room temperature for 5 min. The images was captured by the inverted Olympus IX71 microscope.

To test HO-8910PM cells to grow in an anchorage-dependent manner in three types of hydrogels, 10,000 HO-8910PM cells were prepared in 10 μL drops of RADA16-I hydrogel, Matrigel, and collagen I, respectively, according to 3D cell culture protocols described above except that the scaffold concentrations changed to one tenth of 3D cell culture condition that might facilitate the tumor spheroid formation in hydrogels. The drop of each hydrogel-cell clump was placed in the middle of 96-well cell culture plate well, which was then inverted for the first 30 min of scaffold polymerization, entrapping HO-8910PM cancer cells in the drop and preventing collection of HO-8910PM cancer cells at the bottom. The cell culture plate was then turned right side-up and allowed to polymerize for an additional 30 min in a 37 °C incubator and 200 μL cell culture medium was added to surround the drop carefully and changed every other day. Since HO-8910PM cancer cells had almost consistent growth rate when casted in 3D cell culture plate well, the tumor cells aggregated to form well-defined cell aggregate for 6 days. Though big tumor spheroids were almost grown from the dispersed HO-8910PM cells in normal medium for 15 days. The corresponding images were collected by a common Leica DMILLED microscope at the desiring days.

### Immunohistochemistry

Gel-cell clumps were normally cultured for 7 days as described above and fixed with 4% paraformaldehyde and dehydrated with the gradient ethanol, and then embedded in paraffin, and cut into 5 μm sections. The sections were deparafinized and rehydrated. Endogenous peroxidase activity was quenched by using 40 mL pure methanol mixed with 1.3 mL of hydroxygen peroxide solution for 10 min. The sections were repaired in citrate buffer (pH 6.0) for 10 min, closed with 2% BSA for 30 min, blocking non-specific antigen. The solutions of anti-E-cadherin (1:500), anti-N-cadherin (1:400) and anti-integrin-β1 (1:50) were then added and incubated for 16 h at 4 °C. After 30 min incubation at 37 °C, the horseradish peroxidase-conjugated secondary antibody (1:50) was added and incubated for 60 min at 37 °C. After incubated with antibodies, HO-8910PM cells were stained by hematoxylin developed by 3,3′-diaminobenzidine (DAB) substrate. Gel-cell clumps were washed by PBS buffer and differentiated with 0.1% hydrochloric acid and ethanol (n = 4/each hydrogel scaffold). The staining intensity was quantified by ImageJ software (NIH, Bethesda, MD).

### Western blot assay

On day 6, gel-cell clumps were washed twice with ice cold PBS and lysed in ice cold RIPA lysis buffer (50 mM Tris, 150 mM NaCl, 1% Nonidet P-40, 0.25% sodium deoxycholate, 1 mM EDTA) containing a cocktail of protease inhibitor (0.1 mM PMSF, 5 mg/mL aprotinin, 5 mg/mL pepstatin A, and 1 mg/mL chymostatin)(Sigma-Aldrich, St. Louis, MO, USA) for 3 h on ice. During incubation, cell lysates were vortexed at each 10 min interval and then centrifuged at 26,000*g* for 30 min at 4 °C. The supernatant was harvested to serve as whole cell proteins. Protein concentration was determined by BCA protein concentration kit. Equal protein concentrations from each sample were mixed with Laemmli sample-loading buffer for sodium dodecyl sulfate polyacrylamide gel electrophoresis (SDS-PAGE). After transferred to PVDF membranes (Millipore, bedford, MA, USA) using an Semi-Dry Transfer Cell device (Bio-Rad), incubated with the blocking buffer (5% fat-free milk) for 1 h at room temperature. Blots were reacted with specific primary antibodies in 5% fat-free milk overnight, further incubated with secondary antibodies. The immunoreactive protein patterns were visualized by enhanced chemiluminescence (Thermo Scientific, Pittsburgh, PA, USA) following the manufacturer’s instruction. GAPDH was served as an internal control. Image analysis was quantified with Image J (NIH, Bethesda, MD) and protein band intensities were digitized to indicate molecular expression levels.

### Drug response assay

The chemosensitivity of HO-8910PM cells in 3D culture was confirmed by MTT cell survival assay as described with some modification [32, 42, 43]. Briefly, HO-8910PM cells were resuspended in a final concentration of 5 × 10^4^ cells/mL. An aliquot (20 μL) of HO-8910PM cells were seeded in RADA16-I hydrogel, Matrigel, and collagen I on 96-well microplate for 3 days, respectively. The cell aggregates were formed and different concentrations of cisplatin and paclitaxel (2 μg/mL, 5 μg/mL, 10 μg/mL, 20 μg/mL, 40 μg/mL for cisplatin; 5 μg/mL, 10 μg/mL, 20 μg/mL, 40 μg/mL, 60 μg/mL for paclitaxel) were added to the plate wells, and incubated for 36 h. IC_50_ (50% inhibition concentration) values were measured by a sigmoidal dose-dependent curve fit analysis (OriginPro8.0 software) including conventional 2D cell culture condition. After gel-cell clumps were further incubated with cisplatin and paclitaxel for 3 days, 50 μL cell isolation solutions and 20 μL of MTT (5 mg/mL, Sigma-Aldrich) were added to the cell culture wells. The gel-cell clumps could be easily associated by mechanical blow with a serum tube or pipette. The microplates were incubated at 37 °C for an additional 4 h. And then 100 μL of 20 mM HCl containing 20% SDS was added to each well and incubated for 12 h at room temperature. Dimethyl sulfoxide (DMSO) was added to each well and mixed for 5 min on an orbital shaker. The resulting formazan crystals were extracted from the plate wells with DMSO. The optical density was recorded with a plate reader at 570 nm, which denoted the drug response of chemosensitivity to cisplatin and paclitaxel. HO-8910PM cells grown in 2D 96-well microplates with the same cell number (approximately 1000 cells) were performed to serve as control, but the cell culture time and drug response time were shorted to be 60% to 80% confluence and 2 days, respectively. HO-8910PM cells that received either no drugs or proper drug concentrations were served as the control well. The cytotoxicity was expressed in the form of inhibition rate (%) of viable cells, that was calculated using the formula: ${\text{Inhibition}}\;{\text{rate}}\; ( {\text{\% ) = }}\frac{{1 - A_{mean\;treated\;wells} }}{{A_{mean\;control\;wells} }} \times 100\%$. All MTT assays were repeated three times and quadruplicate samples were performed for each type of hydrogel matrix.

### Statistical analysis

All data were processed in SPSS 17.0 for Windows and used for statistical analysis. Results were presented as mean ± standard deviation (± SD). Statistical significance was determined for experimental data by the unpaired Student’s *t*-test and One-Way ANOVA analysis. Values 0.05 (*) and 0.01 (**) were assumed as significant levels of difference for all assays.

## Results and analysis

### Scaffold characterization

**Fig. 1:**
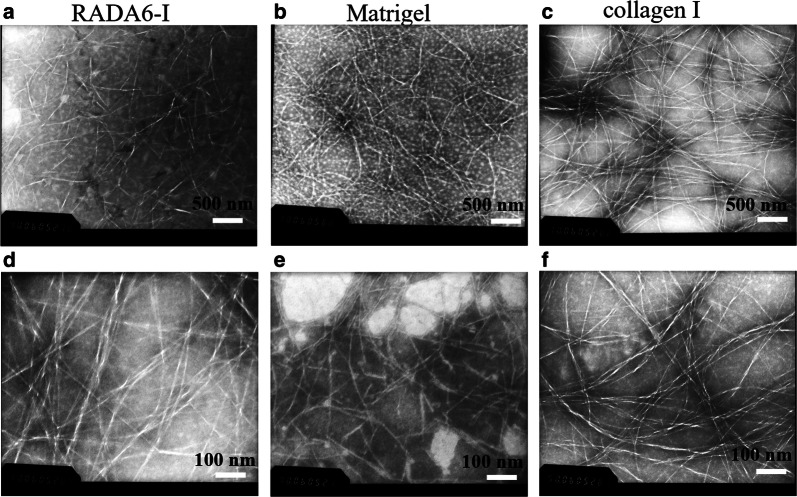
Negatively staining TEM images showed the nanofiber morphology of RADA16-I (**a**, **d**), Matrigel (**b**, **e**), and collagen I (**c**, **f**). RADA16-I, Matrigel, and collagen I were dissolved in 0.1 × PBS solution and self-assembled spontaneously, respectively. Three types of hydrogels all presented a collection of interwoven and disorganized nanofiber networks ultra-microarchitectures in saline solution. Scale bar represented different resolution in TEM images (top panels (**a**–**c**): ×8000; bottom panels (**d**–**f**): ×15,000)

TEM was used to analyze the reticulated nanofiber structure of cell culture scaffold in solution (Fig. 1). In RADA16-I, Matrigel, and collagen I, the nanofiber size and length were obviously different, although they all presented the entangled nanofiber ultra-microstructure in PBS (pH 7.0). At 500 nm scale of TEM images (Fig. 1a–c), the interwoven nanofiber networks were clearly observed in RADA16-I, Matrigel, and collagen I, respectively. The nanofiber alignment in both RADA16-I and collagen I seemed to be well separated and formed more homogenous nanofiber networks than Matrigel (Fig. 1a and c). Matrigel tended to form uneven or irregular and disorganized nanofiber networks architecture (Fig. 1b). At 100 nm high resolution scale of TEM images, both RADA16-I and collagen I formed well separated single nanofiber alignment and had approximately uniform nanofiber size in diameter (Fig. 1d and f). Matrigel showed many short nanofiber scaffold segments and present inconsistent nanofiber size in diameter (Fig. 1e). Moreover, some well-defined nanofibers in TEM images had different widths: 12.5 ± 1.82 nm for RADA16-I peptide, 8.5 ± 1.25 nm for Matrigel, and 11.2 ± 1.76 nm for collagen I, respectively. The single nanofiber width in three types of hydrogels was presumably determined in the range of 7–15 nm. As to nanofiber length, the nanofiber ends weren’t easy to observe because of their entangled, web-like, cross-linked networks architecture. But according to the bar scale, we could approximately suppose that RADA16-I, Matrigel and collagen I formed single nanofiber length with several micrometers in PBS (pH 7.0). Both RADA16-I and collagen I formed longer nanofibers than Matrigel, since most of nanofibers in Matrigel were intertwisted web-like architectures with the stretched moieties on scaffold (Fig. 1b and e). As to the reticulated nanofiber networks in hydrogels, it was evident that both RADA16-I and collagen I formed obviously smoother nanofiber alignment in PBS than Matrigel (pH 7.0).

### Cell viability, proliferation and cell aggregate growth

**Fig. 2:**
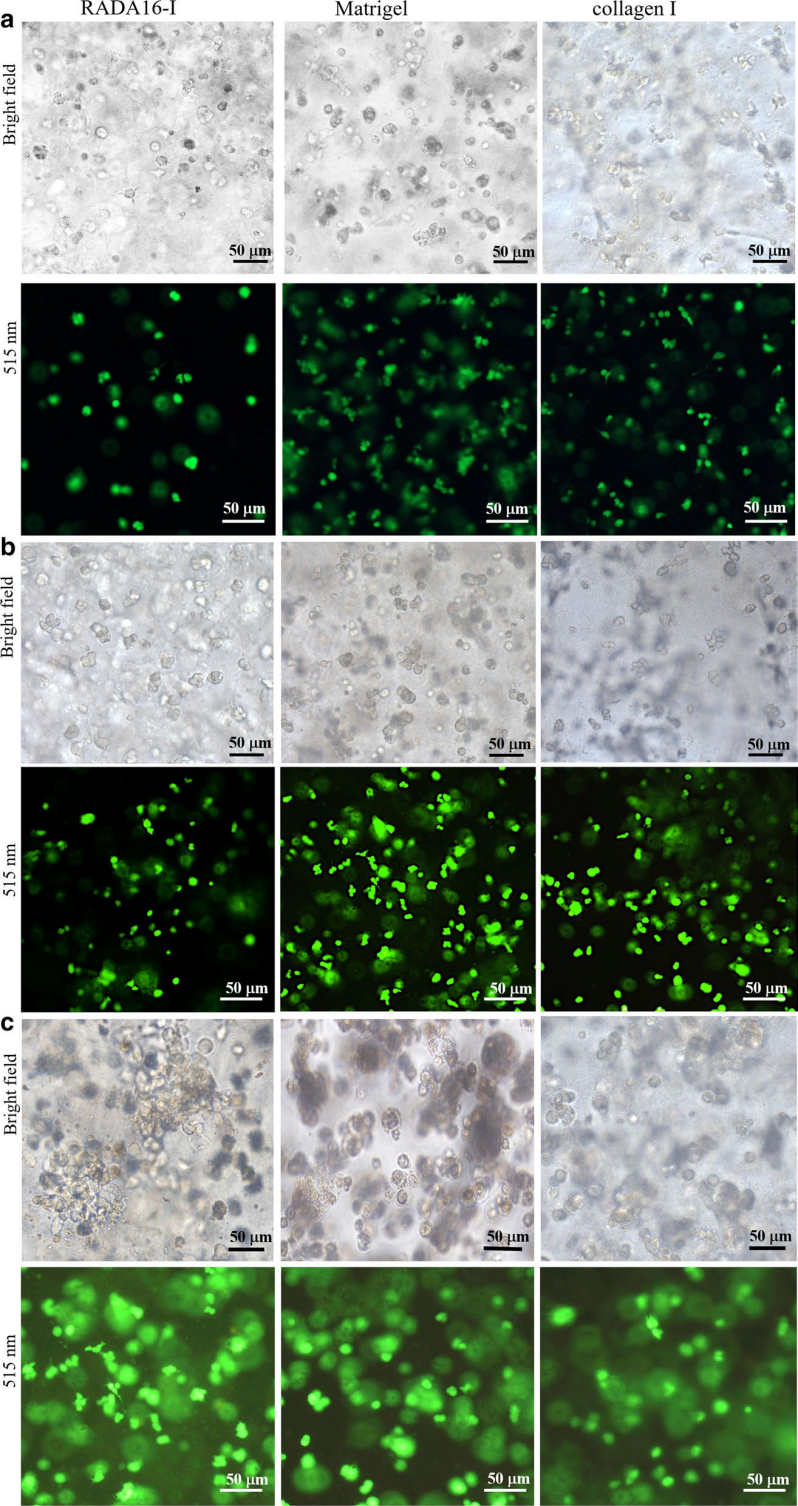
HO-8910PM cell viability and cell proliferation in 3D cell cultures. HO-8910PM cells were encapsulated in RADA16-I hydrogel, Matrigel and collagen I and cultivated for 1 day (**a**), 3 days (**b**), and 6 days (**c**), respectively. Gel-cell clumps were successfully harvested when 3D cell cultures were over at the desired time points. The photomicrographs were taken by phase contrast microscopy (top panels in **a**–**c**). The green fluorescent calcein-AM staining for the living cells was performed to indicate cell viability and robust cell proliferation with distinct cell shapes in all hydrogel volume (bottom panels in **a**–**c**)

As shown in Fig. 2, HO-8910PM cells were regularly cultivated in three types of hydrogels on days 1, 3, and 6. The preliminary cell growth and viable cell fate were observed by phase contrast microscopy and green fluorescent calcein-AM staining (Fig. 2a–c), which confirmed that the majority of tumor cells were viable and active in 3D cell cultures and retained diverse cell growth morphology, such as spherical shape, irregular cell shape, and spindle shape. In the panels by phase contrast microscopy, HO-8910PM cells maintained spherical, irregular or spindle cell shapes (upper panels in Fig. 2a–c). There were no flat or spreading cell shapes. When cultured in hydrogels on days 3 and 6, some HO-8910PM cells formed small cell colonies or cell clusters in the gel-cell clumps (Fig. 2b and c). Some HO-8910PM cells had the elongated spines on cell surface, which primarily evidenced that the nanofiber scaffolds in three types of hydrogels had ECM-like functionality and good biocompatibility for 3D cell cultures in vitro. Furthermore, HO-8910PM cells presented uniform cell distribution in three types of hydrogels. Especially, some HO-8910PM cells stretched out pseudopodia to keep contact with other cells in 3D context (Fig. 2b, c), which envisioned the cell-to-cell adhesion, junctions and interactions in all hydrogel volume. On days 6, some HO-8910PM cells showed irregular cell colonies or cell aggregates in gel-cell clumps (phase contrast panels in Fig. 2c). Interestingly, some HO-8910PM cell colonies or cell aggregates showed the surrounding stretch in the viable cell surface (green fluorescent panels, Fig. 2c), which indicated that HO-8910PM cells formed spatially proper cell aggregates or cell colonies in multicellular morphologies (Fig. 2c) and that RADA16-I nanofiber scaffold in hydrogel presented similar scaffold characteristics with Matrigel and collagen I for 3D cell cultures. It was evident that three types of hydrogels all promoted the cell viability, cell proliferation, and active cell aggregate growth in hydrogels. As to cell recovery, it is a prerequisite for commercial translational usefulness in biomedical fields. As 3D cell-culturing procedure described above, HO-8910PM cell isolation in RADA16-I hydrogel was more convenient and simple process compared to Matrigel and collagen I, since no additional components were added to the cell culture media. The cell colonies, cell clusters, cell aggregates, and tumor spheroid were spontaneously dissociated by mechanical force in medium, since the peptide nanofiber-cell interactions were mediated by non-covalent force with a space-free style and termed as gel-cell clumps.

**Fig. 3:**
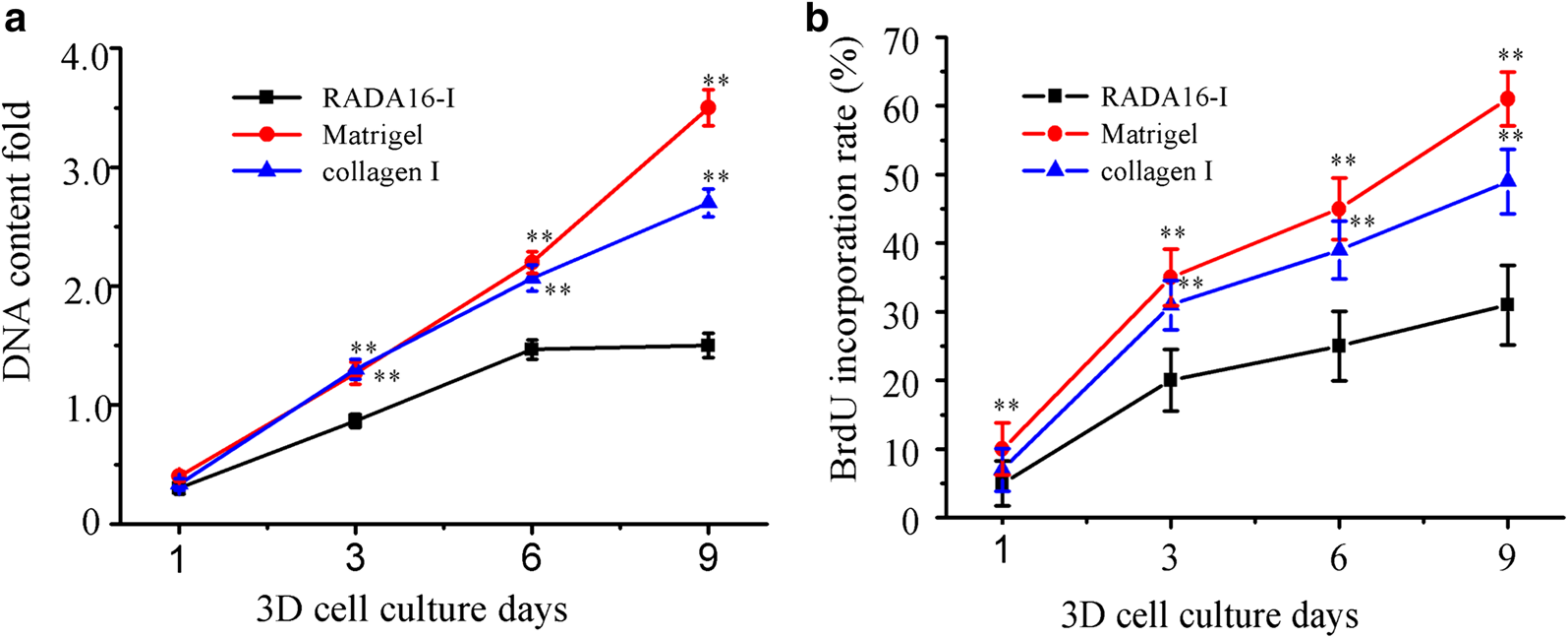
The viable cell proliferation curves of HO-8910PM cells cultured in RADA16-I hydrogel, Matrigel and collagen I on days 1, 3, 6 and 9. **a** HO-8910PM cell proliferation in different hydrogels was calculated from DNA content fold changes at the desired time points. **b** BrdU labelling assay was performed to evaluate the viable cell percentage of HO-8910PM cells seeded in different nanofiber scaffolds, expressed as the cell proliferation rate (%) in three assays (about 200 cells per microscopical view field) on cell culture days 1, 3, 6, and 9. Data showed statistically significant differences (*p < 0.05, **p < 0.01) when compared with RADA16-I hydrogel

In order to evaluate cell viability and active proliferation in hydrogels, as shown in Fig. 3, DNA content of HO-8910PM cells was determined at the desired time points. On day 1, as supposed, there was a relatively low cell proliferation rate in hydrogels at the initial time point. On day 3, HO-8910PM cells cultured in Matrigel and collagen I maintained a rapid growth rate up to day 9. However, HO-8910PM cells cultured in RADA16-I hydrogel only maintained the fast proliferation rate up to day 6 and then presumably retained a flat proliferation rate from day 6 to day 9. Furthermore, since 3D cell culture often maintained very high cell densities in hydrogels, BrdU labeling method was effective to detect the active HO-8910PM cell proliferation in 3D context. When BrdU reagent was incorporated to genomic DNA of 3D-cultured HO-8910PM cells in all hydrogel volume. As shown in Fig. 3b, HO-8910PM cells maintained similar proliferation rate curves with DNA content determination at the desired time points. When BrdU was incorporated, HO-8910PM cell proliferate rate increased up to 20%, 35%, and 31% in RADA16-I hydrogel, Matrigel and collagen I during 3 days 3D cultures, respectively. Cell proliferation rate in Matrigel rose sharply from 45% on day 6 to 65% on day 9. In collagen I, cell proliferation rate increased slowly from 36% to 47% between days 6 and 9, but RADA16-I hydrogel had 25% of cell proliferation rate on days 6 and analogously maintained 31% of slow proliferation rate until days 9, whereas the plateaued cell proliferation rate wasn’t noticed between day 6 and day 9. So, we could suppose that RADA16-I hydrogel still maintained long-term 3D culture growth of HO-8910PM cells by active cell aggregate growth pattern at high cell densities and the viable proliferation capacity in 3D context. As described above, in 9 days 3D cell culture period, the BrdU labelling assay all the time maintained the myogenic proliferation activity of HO-8910PM cells in hydrogels, which suggested that RADA16-I hydrogel might have similar biocompatible capacity with Matrigel and collagen I and actively promoted cell aggregate growth and the viable cell proliferation in 3D cell cultures. These data were in consistent with previous reports by another group [39]. So, due to the viable cell aggregate growth, convenient laboratory usefulness, high cell proliferation rate and preferable physicochemical characteristics, RADA16-I hydrogel was well-suited to serve as biomimetic nanoscale scaffolds and develop the adaptable 3D cell models in vitro by current bioengineering cell technologies.

### Cell aggregate growth and cell vitality

**Fig. 4:**
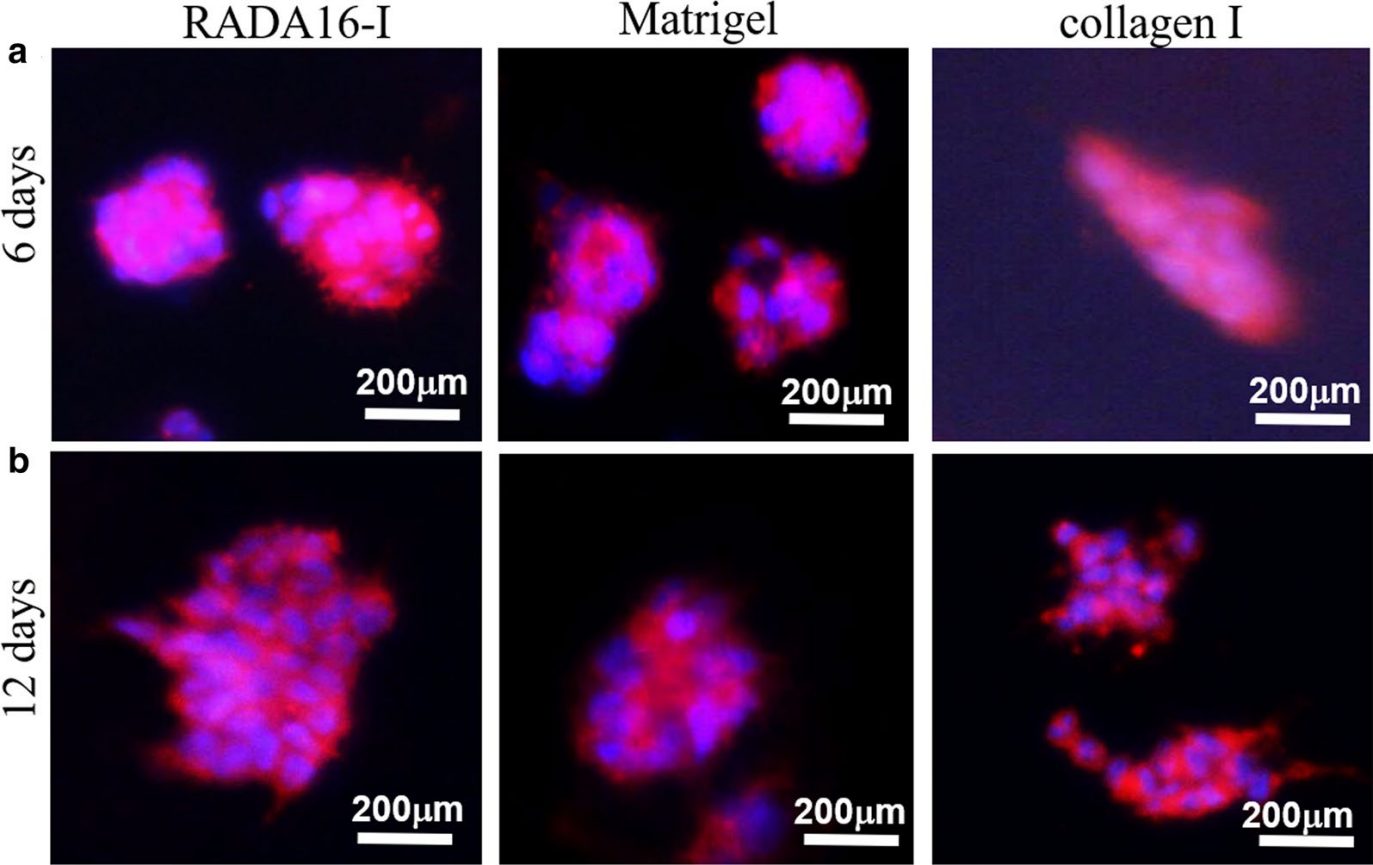
Cell-to-cell interactions and geometry arrangements of HO-8910PM cells in gel-cell clumps. HO-8910PM cells formed MCTS in three types of hydrogel matrices on days 6 (**a**) and days 12 (**b**). Gel-cell clumps of HO-8910PM cell line in RADA16-I, Matrigel and collagen I were harvested on day 6 and day 12. Immunofluorescence assay was performed to indicate cell-to-cell interactions and geometry arrangements in 3D cell culture. Red indicated F-actin and blue indicated DAPI-stained cell nuclear. Immunofluorescence images were obtained by an inverted Olympus IX71 microscope. The blue nuclear staining showed HO-8910PM cell arrangement in MCTS and the red staining surrounding blue nuclear envisioned the cell-to-cell adhesion or intercellular junction in MCTS

As shown in Fig. 4, when cultured for days 6, HO-8910PM cells grew and aggregated to form cell colonies or cell clusters. Some big cell colonies were chosen for F-actin localization, which showed that HO-8910PM cells cultured in RADA16-I hydrogel and Matrigel were spherical cell agglomeration in 3D context and the cellular nuclei were well organized and regularly arranged within cell colonies or cell clusters by the spherical shapes in morphology (left and middle panels in Fig. 4a). HO-8910PM cells cultured in collagen I had the elongated cell agglomeration in 3D context (right panel in Fig. 4a). When cultured for 12 days in hydrogels (Fig. 4b), HO-8910PM cells in RADA16-I hydrogel formed multicellular tumor spheroid (MCTS) with uniform cell distribution approximately (left panel in Fig. 4b). HO-8910PM cells in collagen I formed cell stretches or long cell strips in MCTS in 3D culture (right panel in Fig. 4b), while HO-8910PM cells in Matrigel formed several MCTS in 3D culture (middle panel in Fig. 4b). So, when cultured in RADA16-I hydrogel and Matrigel for a long-term period, HO-8910PM cell nuclei location indicated that MCTS maintained the spherical shapes in morphology and had regular cell alignment in 3D context. When cultured in collagen I, cell nuclei location indicated long cell strips or irregular cell alignment in 3D context. These results suggested that HO-8910PM cells formed distinct cell aggregate growth patterns, such as the MCTS, cell colonies, cell strips, and cell clusters, which represented the biomimetic cell-to-cell adhesion, junction or HO-8910PM cell-nanofiber matrix interactions in 3D cell culture.

As to cell metabolism, in RADA16-I hydrogel, the uniform cell distribution in big MCTS indicated that HO-8910PM cells had enough nutrients and oxygen to maintain normal cell metabolism and cell vitality (lef panel in Fig. 4b). In Matrigel, hollow MCTS indicated that hypoxia area appeared in the center position of MCTS and HO-8910PM cells possibly showed apoptotic phenotype or ankosis phenotype in 3D cell culture of Matrigel for the long period (middle panel in Fig. 4b). In collagen I, the slender cell aggregate in cell strips or multiple cell stretches were similar to the mesenchymal cell vitality with irregularly organized cell nuclei because collagen fibers limited big MCTS formation in 3D context (right panels in Fig. 4a and b). With the increasing cell culture time, HO-8910PM cells cultured in RADA16-I hydrogel still showed radially asymmetrical cell aggregate architecture in MCTS (left panel in Fig. 4b). So, it was evident that RADA16-I hydrogel was useful to maintain the native cell–cell adhesion or junction and cancer cell-nanofiber matrix interactions that were intrinsic phenotype presentation in tumor metastasis procedure in vivo or malignant cell behaviors in 3D culture [5]. Moreover, HO-8910PM cells cultured in RADA16-I hydrogel and Matrigel had much large cell aggregates, cell colonies or cell clusters and presented minimal single cell presentation (Figs. 2c, 4a and b). Surely, these cell colonies, cell strips, cell clusters or MCTS formed as a result of active cell proliferation, viable cell-to-cell adhesion, proper cancer cell-ECM interactions, and high cell vitality in hydrogels. These cell aggregate morphologies represented the different cell growth patterns of HO-8910PM cells related to the cell vitality in three types of hydrogels for a long-term cell culture period, which indicated that the nanofiber microenvironments in hydrogels provided proper physicochemical properties and mechanical stiffness for 3D cell culture in vitro.

**Fig. 5:**
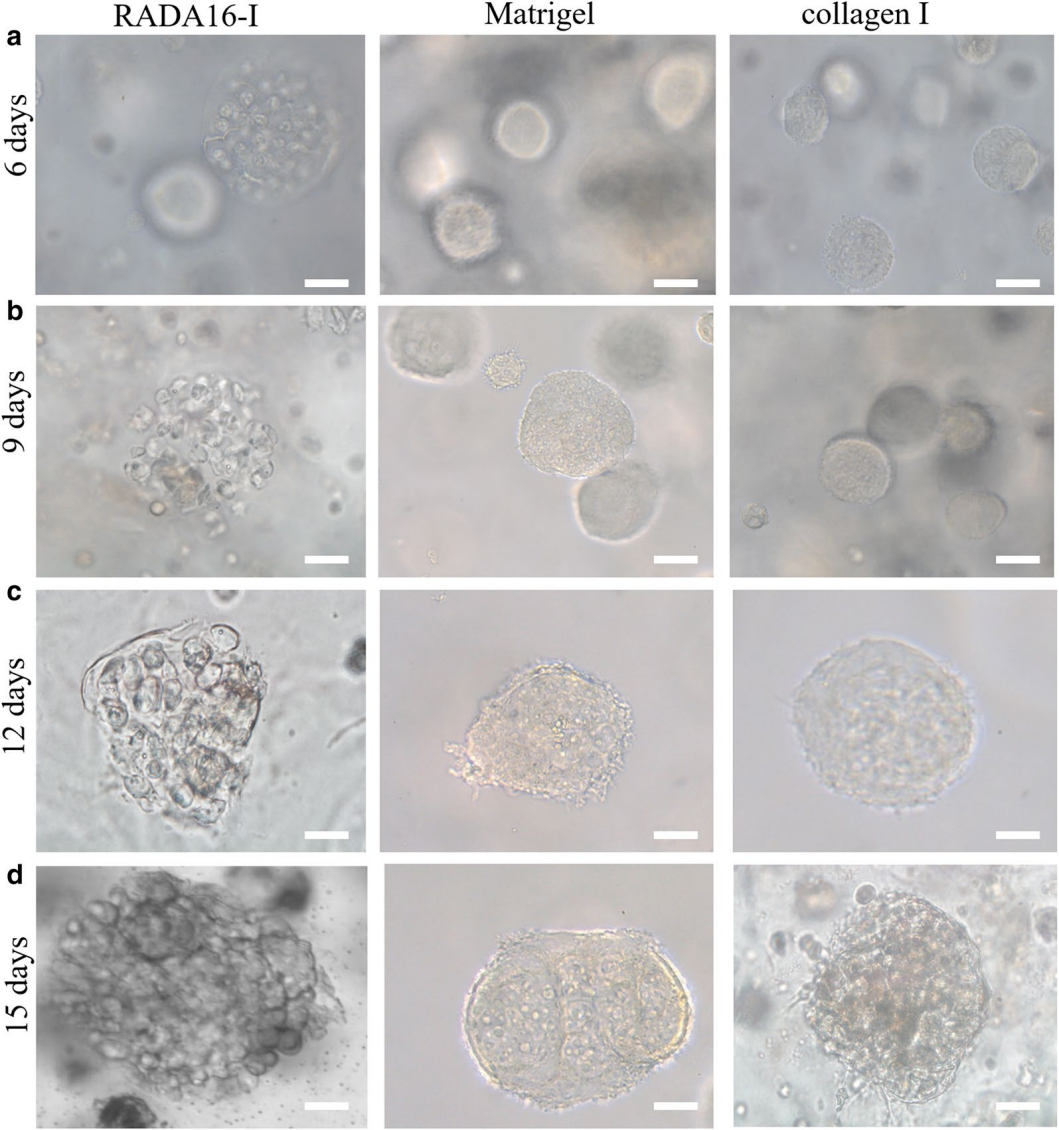
Representative images of MCTS for cell aggregate growth patterns in one tenth scaffold concentration and cell density when cultured for 6, 9, 12, and 15 days, respectively. HO-8910PM cells formed MCTS in three types of hydrogels and maintained different cell aggregate growth patterns in 3D cell culture for 15 days. MCTS was imaged through bright-field phase contrast microscopy. Phase contrast images were some representatives for HO-8910PM cells harvested at the desired time points. Scale bar was 100 μm

Except of high cell density pay-load in 3D cell cultures, when both the scaffold concentration in hydrogels and cell seeding density decreased to one tenth for 3D cell culture as described in method, MCTS was generated in three types of hydrogels to indicate the cell growth patterns in 15 days cell culture period (Fig. 5). The diluted scaffold concentration and cell density facilitated the MCTS formation in 3D cell culture. Due to the access of nutrients in nanofiber microenvironment for 3D cell culture, HO-8910PM cells presented the distinct cell aggregate growth patterns in 3D cell culture. On 6 days cell cultures, HO-8910PM cells in cell colonies gradually expanded and formed big MCTS in three types of hydrogels, which indicated that the robust cell growth and viable propagation were established for 3D cell culture in hydrogels (Fig. 5a). For 3D cell culture in RADA16-I hydrogel for 9 days, HO-8910PM cancer cells formed big MCTS so that we could identify the dotted cell clusters dispersed and stretched out of the hydrogel surface, which continued to day 12 and day 15 to maintain big MCTS growth pattern (right panels in Fig. 5c and d). However, HO-8910PM cells in RADA16-I hydrogel started to change the spherical MCTS morphology in 3D cell culture for day 9, some HO-8910PM cells started to expose evenly on the external hydrogel surface and localize on the edge of MCTS, which was clear on day 12. When cultured on day 15, many HO-8910PM cells were exposed out of the hydrogel surface in all hydrogel volume. During 3D cell culture in Matrigel from day 6 to day 15 (middle panels in Fig. 5), HO-8910PM cells maintained the consistent MCTS growth pattern all along and formed compact MCTS in morphology. For 3D cell culture in collagen I from day 6 to day 15 (right panels in Fig. 5), HO-8910PM cells maintained initial and small cell aggregate growth pattern in 3D cell culture for 6 days. HO-8910PM cells started to change the MCTS morphologies from day 9 to day 15. On day 9, the MCTS present irregular and spherical cell aggregates in morphology; on day 12, some HO-8910PM cells were exposed out of hydrogel surface and a few HO-8910PM cells stretched out of the hydrogel surface; on day 15, more and more HO-8910PM cells spread out of the MCTS edge and showed irregular and big MCTS in morphology. These results collectively indicated that HO-8910PM cells grew better in RADA16-I hydrogel, Matrigel, and collagen I and that RADA16-I hydrogel had analogous ECM-like scaffold affinity to Matrigel for cell aggregate growth patterns in nanofibrous cell microenvironment. It was evident that RADA16-I hydrogel was a robust, adaptable, and competent 3D cell culture platform, which raised the viable cell aggregate growth of HO-8910PM cells in a biomimetic nanofiber cell microenvironment.

### Cell adhesion protein expression

**Fig. 6:**
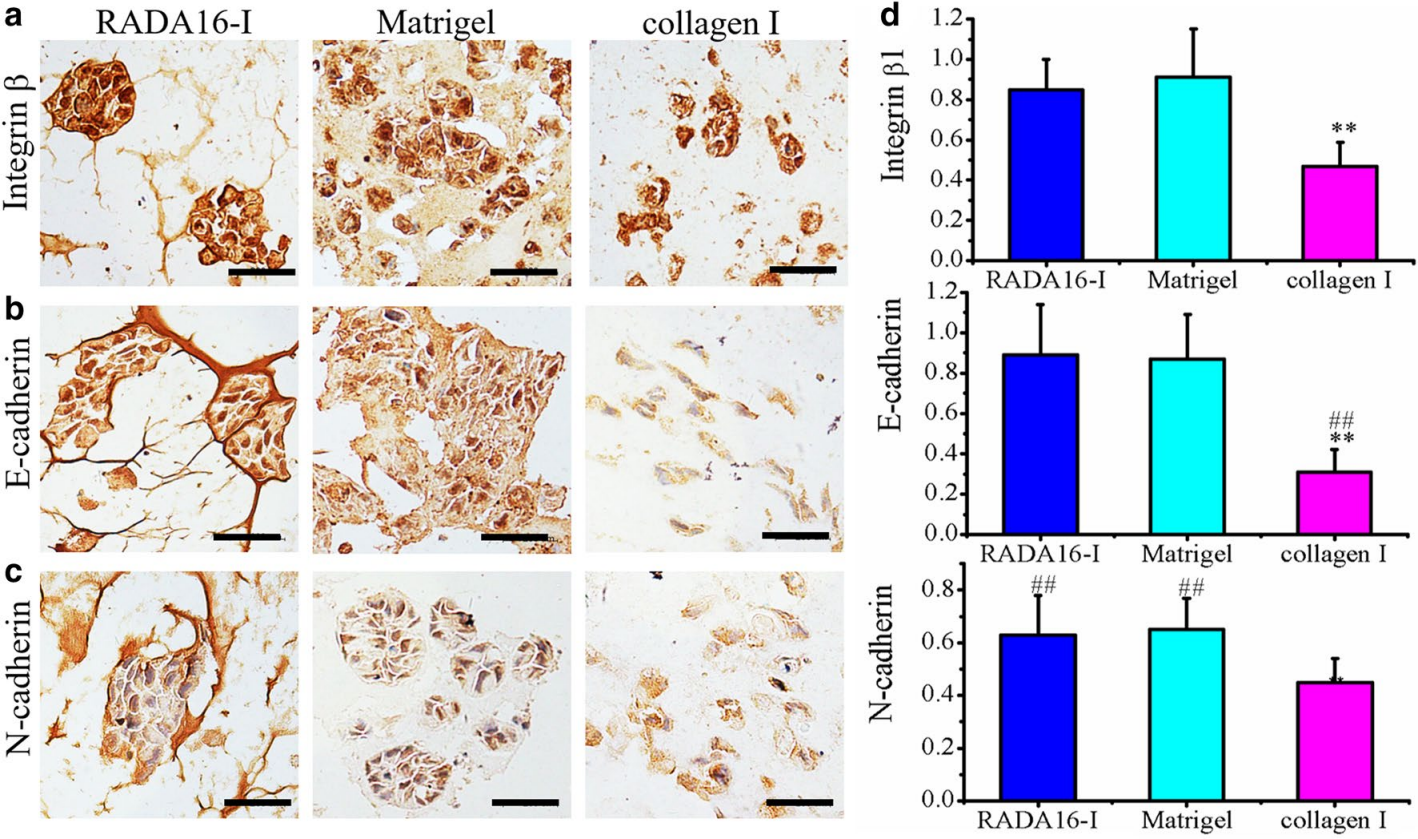
The cell distribution and molecular expression of integrin β1 (**a**), E-cadherin (**b**) and N-cadherin (**c**) in HO-8910PM cells cultured in RADA16-I hydrogel, Matrigel and collagen I for 7 days. The brown color indicated the viable proliferation cells, which revealed a solid tumor-like tissue architecture and MCTS formation that was stained by hematoxylin in sections. **d** Relative quantification of protein expression levels in RADA16-I hydrogel, Matrigel and collagen I. Immuno-expression of integrin β1, E-cadherin and N-cadherin was quantified as an index of positively staining area over total hematoxylin-staining section area in gel-cell clumps. **P < 0.01 denoted hydrogel matrix compared; ^##^P < 0.01 denoted cell adhesion molecules compared. Scale bars were 200 μm

**Fig. 7:**
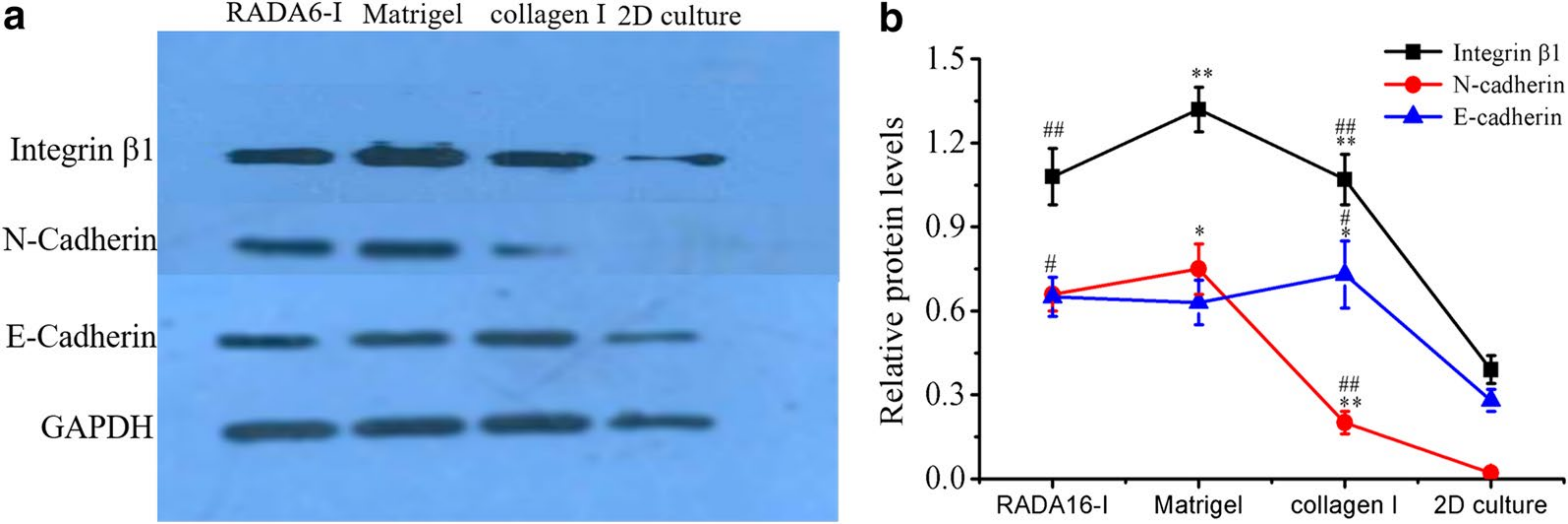
Western blot analysis of key cell adhesion proteins in HO-8910PM cells cultured in RADA16-I hydrogel, Matrigel and collagen I for 7 days. **a** Immunoblot showed qualitative molecular expression of integrin β1, E-cadherin and N-cadherin in different hydrogel biomaterials. **b** The curve graph indicated the densitometry quantitation of integrin β1, E-cadherin and N-cadherin protein expression levels normalized to GAPDH. Data represented the mean ± SD in three independent assays and showed statistical difference. *P < 0.05 indicated significant difference and **P < 0.01 indicated higher significant difference when compared with RADA16-I peptide scaffold. ^#^P < 0.05 indicated significant difference and ^##^P < 0.01 indicated higher significant difference when compared with Matrigel

RADA16-I peptide scaffold is synthesized chemically and has been studied well for many cell types in tissue engineering or regenerative medicine. Matrigel and collagen I have also been demonstrated to be well-suited for use as cell scaffolds in a variety of cell types, including cancer cells. Integrin β1 together with E-cadherin and N-cadherin made the epithelial tissue organization in virtue of their involvement in cell–cell adhesion, cell aggregate formation or cell-ECM interactions [44, 45]. Although some reports studied the distinct cell–cell adhesion or cell-hydrogel matrix interaction in RADA16-I hydrogel and Matrigel or collagen I [39–41, 46], the molecular expression of cell adhesion proteins was altered in different MCTS formation since the distinct hydrogel matrices conferred different tumor cell-ECM interactions in 3D context. As shown in Fig. 6, by immunohistochemical staining, the molecular expression of integrin β1, E-cadherin and N-cadherin was preliminarily identified in ovarian cancer HO-8910PM cell line when cultured in three types of hydrogel biomaterials for 7 days. When normalized to single cell surface on section, HO-8910PM cells cultured in RADA16-I hydrogel and Matrigel showed significantly higher expression of integrin β1, E-cadherin, and N-cadherin when compared with collagen I (P < 0.01), and there was no significant difference between RADA16-I hydrogel and Matrigel (Fig. 6a–c), which indicated that RADA16-I hydrogel presented similar cell scaffold characteristics with Matrigel in 3D cell culture. In both RADA16-I hydrogel and Matrigel, integrin β1 and E-cadherin showed greatly higher expression than N-cadherin. While in collagen I, integrin β1 and N-cadherin showed significantly higher expression than E-cadherin and there was no significant difference of N-cadherin expression between RADA16-I hydrogel and Matrigel. Since integrin β1 and E-cadherin were molecular markers to indicate cellular adhesion ultra-microstructure, including cell–cell adhesion, cell–cell junction, it was evident that MCTS formation in RADA16-I hydrogel and Matrigel retained the epithelial-like phenotype and proper cell-ECM integrity in 3D cell cultures and maintained in vivo-like cell tissue architectures, which was also demonstrated by other reports [47, 48], while E-cadherin/N-cadherin predominantly designated epithelial-to-mesenchymal transition and enabled malignant cancer cell adhesion, junction, migration and invasion in 3D context [49, 50]. These results confirmed that 3D culture of HO-8910PM cells in RADA16-I hydrogel formed proper cell–cell adhesion or compact cell-ECM interactions that was similar with 3D cell culture properties in Matrigel. So, HO-8910PM cell–peptide nanofiber adhesions were likely analogous to cell–matrix interactions in Matrigel. MCTS formation initially involved in the formation of loose cell aggregates via integrin-mediated adhesion followed by the expression of E-cadherin and the formation of compact tumor spheroids through hemophilic cadherin–cadherin interactions [51]. By further quantitative blot analysis of key proteins related to MCTS formation in 3D cell cultures (Fig. 7), Integrin β1 almost showed the same similar expression intensity as E-cadherin in RADA16-I hydrogel, collagen I, and 2D cell culture, except that integrin β1 had significantly higher expression in Matrigel. E-cadherin and N-cadherin expression detected by western blotting were approximately similar to those results by immunohistochemical assay (Fig. 6b and c) except that integrin β1 and E-cadherin showed significantly high expression in collagen I, which is different in immunohistochemical assay (right panels in Fig. 6). Moreover, N-cadherin had lower expression in RADA16-I hydrogel than Matrigel and very low expression in collagen I and even no expression in 2D cell culture. Collectively, these results indicated that HO-8910PM cells cultured in RADA16-I hydrogel and Matrigel formed proper cell–cell contact or intercellular junction and spatially compact MCTS than 3D-cultured HO-8910PM cells in collagen I.

### Chemosensitivity assay

**Fig. 8:**
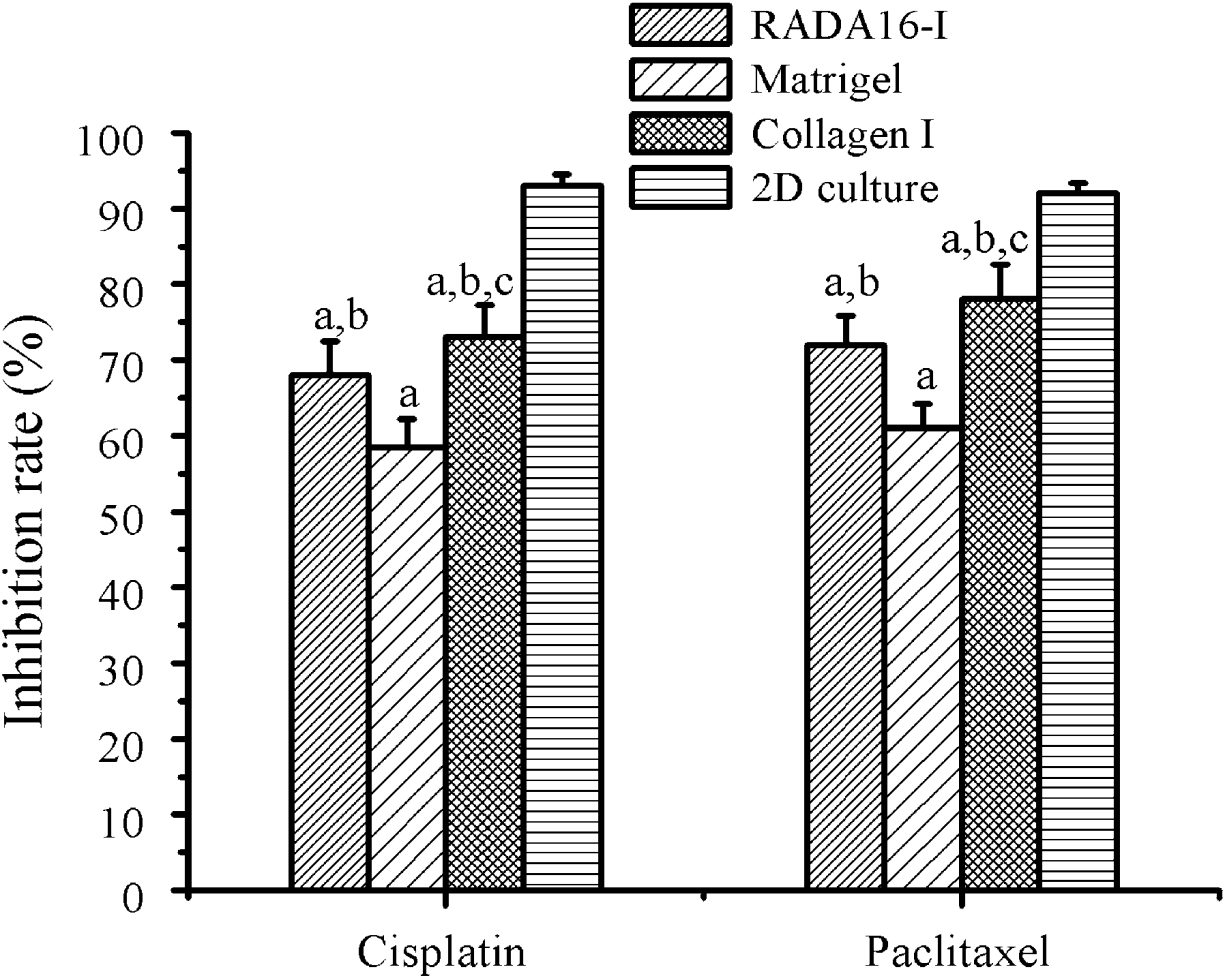
Drug responses of chemosensitivity assay in HO-8910PM cells cultured in gel-cell clumps and common 2D flat cell plates by clinically first-line chemotherapeutic agents (cisplatin and paclitaxel). The precultured cancer cells in three types of hydrogel biomaterials were evaluated by the end point assay for cell survival. ^a^(P < 0.01) compared with that of 2D cell culture model. ^b^(P < 0.01) compared with that of Matrigel. ^c^(P < 0.01) compared with that of RADA16-I hydrogel

Both cisplatin and paclitaxel were first-line therapeutic drugs for clinical ovarian cancer treatment. Two reagents had the different therapeutic mechanism: cisplatin interacted with DNA to interfere with DNA repair, while paclitaxel disrupted microtubules essential to mitosis [52]. As to IC_50_ values in Table 1, HO-8910PM cells cultured in three types of hydrogels conferred the distinct chemosensitivity responses to cisplatin and paclitaxel when compared with 2D flat culture on cell plates at presumably equal cell numbers. For both cisplatin and paclitaxel, HO-8910PM cells in Matrigel showed highest IC_50_ values when compared with collagen I and RADA16-I hydrogel and there wasn’t significant difference between collagen I and RADA16-I hydrogel. In details, the IC_50_ values of cisplatin in RADA16-I and collagen I were 2.23-folds and 2.31-folds higher than the IC_50_ values of HO-8910PM cells cultured in 2D cell culture. When HO-8910PM cells were cultured in Matrigel, the IC_50_ of cisplatin was 3.45-folds higher than HO-8910PM cells cultured in 2D flat cell plate. When paclitaxel was used for the chemosensitivity assay in 3D context, the IC_50_ values of HO-8910PM cells in RADA16-I hydrogel, Matrigel, and collagen I are 1.58-, 1.51-, and 1.80-fold higher than HO-8910PM cells in 2D cell culture, respectively. These results possibly suggested that 3D HO-8910PM cell cultures in RADA16-I hydrogel, Matrigel and collagen I were useful to characterize clinical drug responses in cell models, which significantly decreased the chemosensitivity of HO-8910PM cells for both cisplatin and paclitaxel. Furthermore, we compared the inhibition rate of HO-8910PM cells cultured for 3 days in three types of hydrogel biomaterials with the approximately equal cell numbers in 2D cell culture (Fig. 8). The inhibition rates of cisplatin in RADA16-I hydrogel, Matrigel, and collagen I were 68 ± 4.5 (%), 58.5 ± 3.7 (%), and 73 ± 4.2 (%), respectively. However, paclitaxel in RADA16-I hydrogel, Matrigel, and collagen I conferred the inhibition rates of 72 ± 3.9 (%), 61 ± 3.2 (%), and 78 ± 4.6 (%), respectively. Both cisplatin and paclitaxel showed significantly lower inhibition rates in 3D cell cultures compared with 2D cell culture with the equal cell numbers, which had the inhibition rates of 93 ± 1.5 (%) and 92 ± 1.2 (%) for cisplatin and paclitaxel, respectively. The inhibition rate of cell viability in RADA16-I hydrogel was slightly higher than Matrigel and relatively lower than collagen I, which indicated that RADA16-I hydrogel might provide physiologically relevant cell microenvironment for 3D cell culture and was well-suited for developing the drug chemosensitivity response platform for preclinical or clinical drug screening assay in vitro by 3D cell cultures. Although the inhibition rate was increased with the increase in treatment time and drug concentration in both gel-cell clumps and 2D flat cell culture, in approximately equal cell number conditions, these results firmly indicated that three types of hydrogel biomaterials could properly maintain the preferable cell vitality phenotype to present the cell chemosensitivity detection of drug responses in 3D cell culture in vitro.

**Table 1:** The IC_50_ values in chemosensitivity assay in 3D cell cultures of HO-8910PM cells

Cell culture groups	Cisplatin		Paclitaxel	
	Conc. (μg/mL)	IC_50_ (μg/mL)	Conc. (μg/mL)	IC_50_ (μg/mL)
Matrigel	1–20	6.79^a^	2–50	12.44^a^
Collagen	1–20	4.87^a,b^	2–50	10.26^a,b^
RADA16	1–20	4.70^a,b^	2–50	10.72^a,b^
2D culture	1–20	2.11	2–50	6.79

^a^(P < 0.01) compared with 2D cell culture

^b^(P < 0.01) compared with Matrigel

## Discussion

In the present study, an activatable 3D cell culture of HO-8910PM cell was performed in RADA16-I hydrogel, Matrigel and collagen I, which represented current state-of-the-art cell models especially in biomimetic and advanced RADA16-I designer peptide hydrogel. In the nanofiber cell microenvironments of RADA16-I hydrogel, HO-8910PM cell shapes, the cell viability, cell proliferation rate, cell aggregate growth patterns, the cell vitality, the qualitative expression of cell adhesion molecules, and cellular chemosensitivity detection of clinical therapeutic drugs were comprehensively compared with the counterparts in Matrigel and collagen I, which were current cell culture gold standard for mimicking the physiologically native microtumor tissues by bioengineering cell technology [53–55].

For robust 3D cell cultures, RADA16-I peptide firstly assembled to form the reticulated nanofiber networks with noncovalent interactions in a molecular self-assembly manner. The hydrogelation was induced directly by mixing peptide solution with the isosmotic sucrose solution, which not only avoided the complicated chemical cross-linking reactions, but also utilized nontoxic component generally used in cell culture assay, which intrinsically mimicked much more physiological milieu and isotonic nanofiber cell microenvironment to maintain active cell growth, which was a high-profile cell technology in 3D cell culture assay. Usually, collagen I hydrogelation was slow and must been placed on ice to prevent premature hydrogelation. The pH value of the gel solution had to adjust to neutral condition for viable cell survival and proper cell growth. Collagen fibrils self-assembled into the bundled fibrils with diameters ranging from 12 to 120 nm scale and the collagen fibrils further crosslinked into 3D microporous matrix by the physical association pattern [56–58], which obviously increased the complexity of cell encapsulation in 3D culture assay. Matrigel contained major components of basement membrane in mouse sarcoma and a variety of growth factors. It was widely used to culture many types of cells [3, 59]. Since it could influence cell behaviors and phenotype presentation due to complex ECM proteins and growth factors, Matrigel might not be an appropriate candidate for relative cell signaling and special cell behavior studies. In RADA16-I hydrogel, Matrigel, and collagen I, the cell seeding protocols in RADA16-I hydrogel and Matrigel were accomplished by directly mixing matrix scaffold with culture medium without any pH or temperature change. Therefore, RADA16-I hydrogel was a kind of enough soft, highly hydrated and mechanically tunable nanofiber scaffold, which was versatile and adaptable 3D cell culture in bioengineering nanotechnology by a user-directed manner. Ashwortha et al. adequately addressed that designer self-assembling peptides were chemically synthesized, flexibly tunable and readily adaptable building blocks for hydrogel formation and represented the appropriate intermediates between natural and synthetic nanomaterials [60]. Many investigators comprehensively reported the self-assembly behaviors of (RXDX)_4_ in solution when exposed to pH value, ionic type, and assay time [2, 29, 61–63]. Similarly, some integrative high resolution methodology was utilized to identify ultra-microstructure of designer peptide hydrogel, Matrigel or collagen I at nanometer scale [57, 59, 64–66]. Following the pioneering work by Koutsopoulos and others [38, 41, 46], a variety of cell types were seeded in RADA16-I hydrogel for 3D cell cultures in vitro. Herein, Matrigel or collagen I resulted in hydrogels with rheological properties resembling those of RADA16-I hydrogel. The nanofiber networks and scaffold size or width in RADA16-I hydrogel, Matrigel and collagen I were well studied and analyzed, which were basically consistent with previous results as well [30, 67]. So, the reticulated and web-like nanofiber cell microenvironments were key to establish robust 3D cell cultures in hydrogels.

The viable 3D cell culture was an important tool for us to develop the drug response assay for preclinical or clinical drug screening platform in cell chemosensitivity detection. Our results showed that RADA16-I hydrogel maintained viable cell proliferation and active cell growth of HO-8910PM cells and showed good biocompatibility that was similar with Matrigel and collagen I. During 3D cell culture procedure, HO-8910PM cells showed a sustained and rapid growth in RADA16-I hydrogel, Matrigel, and collagen I, except of a plateaued proliferation rate in RADA16-I hydrogel after 6 days 3D cell culture, which was consistent with our previous reports [39–41]. This was due to Matrigel and collagen derived from animal materials, which contained biological components such as growth factors and potential attachment site, that supported viable cell proliferation and active cell growth in a long cell culture period [5, 59]. RADA16-I hydrogel belonged to fully chemically synthetic nanomaterial and had single ingredient composition, which limited too high proliferation rate in 3D cell culture [13, 68]. As to nanofiber microenvironments in three types of hydrogels, HO-8910PM cells all formed cell aggregates, cell colonies, cell clusters, cell strips and MCTS and represented diverse 3D cell growth patterns in hydrogels. So, RADA16-I hydrogel had the analogous physicochemical properties or mechanical stiffness to Matrigel and collagen I, except that HO-8910PM cells seeded in collagen I to form small cell clusters with mesenchymal cell alignment and cell nuclei organization after 6 days 3D culture (Fig. 4a and b). So, RADA16-I hydrogel was more analogous to Matrigel when compared with collagen I. In addition to the simple ingredients in RADA16-I hydrogel without impurities and the impact of animal-derived pollution or immunogenic issues [14, 68], RADA16-I hydrogel represented a versatile type of bioinspired and high-profile matrix scaffold to form nanofiber cell microenvironments for 3D cell culture in vitro.

For MCTS formation, HO-8910PM cells were consistent with other cancer cell lines in different nanofiber densities and cell culture procedures [60, 63, 69]. The compact cell packing in MCTS was particularly beneficial for the viable survival of ovarian cancer cells either in 3D culture or in the metastatic procedure in vivo. By forming MCTS, ovarian cancer cells could resist the apoptosis phenotype and anoikis, that was a specialized form of apoptosis when cell adhesion to the correct substrate was disrupted [48, 70]. Both integrin and cadherin were involved in robust cell aggregate growth and MCTS formation at molecular levels. Epithelial cells aggregation was primarily mediated by E-cadherin and cell aggregation of more aggressive malignant cells was directed by integrin-ECM interaction. When cultured in RADA16-I hydrogel, Matrigel, and collagen I for 7 days, immunohistochemistry and immunoblot confirmed that HO-8910PM cells in RADA16-I hydrogel and Matrigel expressed high levels of integrin β1, E-cadherin and N-cadherin, while HO-8910PM cells in collagen I expressed relatively low levels of integrin β1, E-cadherin and N-cadherin. Especially, HO-8910PM cells in RADA16-I hydrogel showed slightly low expression of N-cadherin, which was associated with the formation of loose compact MCTS, in contrast, integrin β1 was involved in compact MCTS and the aggressive malignant cell aggregates or mesothelial cells [9, 48, 71]. Our results were consistent to previous reports which formed large MCTS with tight intercellular junctions in ovarian cancer cells [71–73], which suggested that RADA16-I hydrogel provided HO-8910PM cells with well-suited cell microenvironments for 3D cell cultures in vitro. As to other intercellular communication and the signaling pathways related to cell-ECM interactions, when cultured in RADA16-I hydrogel, HO-8910PM cells maintained the intrinsic phenotypes development, active cell differentiation, and the potential tumor progression in 3D cultures in vitro for 15 days. So, RADA16-I hydrogel had proper mechanical stiffness and biomimetic extracellular milieu for 3D cell culture. As demonstrated by other reports [60, 69], compared with Matrigel and collagen I, two types of naturally-derived biopolymer components with good ECM properties, RADA16-I hydrogel was a more proactive alternative of artificial hydrogel matrix for 3D cell culture in vitro.

In anticancer drug discovery, many drugs exhibited high cytotoxic effects of cancer cells in vitro and lost efficacy in vivo in clinical trials, which was mainly derived from chemo-resistant recurrence related to TME involved in cell-ECM interactions. It was evident that the anticancer drugs screening on a platform using 2D-cultured cell lines was unable to precisely select clinically-active compounds. 3D cell cultures had the potential for better simulating the TME in vivo and might help us bridge the gap between 2D cell culture and human being’s tissue in vivo, including maintaining native cell phenotype and in vivo cell functionality [42, 74, 75]. As illustrated in Table 1, the IC_50_ values of cisplatin and paclitaxel, when tested in RADA16-I hydrogel, Matrigel, and collagen I, were significantly higher than those in 2D-cultured HO-8910PM cells. In other cancer types, the tumor cells in 3D culture showed much higher resistance to chemotherapeutics or resulted in the cell phenotype reversion [39, 70]. Our results indicated that 3D-cultured HO-8910PM cells were much more resistant to cisplatin and paclitaxel than their 2D flat monolayer counterpart. When compared with Matrigel and collagen I, RADA16-I hydrogel had more facile cell viability and flexible scaffold deformability at microscale by space-free style and the cell recovery from RADA16-I hydrogel was easy to perform other cell assays. So, 3D cell culture in RADA16-I hydrogel was a simple, robust and flexible option in laboratory usefulness and biomedical translational possibilities, which were demonstrated by a variety of cell types [33, 69]. Here, our results were consistent with the research involved in other type of cancer cells [48, 76]. Designer RADA16-I peptide hydrogel showed in vivo-like ECM niche for tumor cell growth and recapitulated the intricacies of native tissue in vivo. To customize precise 3D cell models, an available approach aiming at controlling and defining TME was to develop artificially biomimetic cell scaffolds, which mimicked 3D ECM cues in a cell type-specific manner. HO-8910PM cell was a highly metastatic human ovarian cancer cell line, which was useful to dissect extracellular factors involved in tumor metastasis or anti-metastatic agents [77, 78]. The future assays in cell type-specific 3D cultures were to customize more physiologically-relevant hydrogels for drug discovery and tumorigenesis or tumor progression. Especially, some specific tumor tissue types such as ovarian cancer, pancreatic cancer, prostate cancer, et al., of which the physiologically-relevant 3D cell cultures in vitro not only poised an enormous challenge but remained an ongoing need in place of human being’s assay and laboratory animal models by bioengineering nanotechnology.

The MTT end point assay, which evaluated total tumor cell viability, was more appropriate than the proliferating-cell end point assay, since tumor cells were maintained in the resting stage of the cell cycle [69]. For 3D cell culture in hydrogels, HO-8910PM cells pursued to perform the extreme drug resistance assay to guide clinically-therapeutic drug screening in vitro, especially for advanced ovarian cancer patients, of which the tumor cells in ascites was easy to collect from patients in hospital. Based on individual cell functionality and genetic differences, one simple assay setting-up or well-controlled 3D cell cultures in hydrogels might been applied to determine the patient prognosis or select alternative chemotherapeutic drugs, enabling chemotherapeutic regimens to be personalized as soon as possible. In the drug response assay of chemosensitivity detection, the cut-off values of HO-8910PM cells were compared not only by IC_50_ values but also by the inhibition rate (percentage), which presented relatively quantitative assessment for drug responses and high inhibition rates, although these values in RADA16-I hydrogel were obviously higher than Matrigel. One report evidenced that the assay accuracy was 82.8% or beyond [42]. Another chemosensitivity assay of 11 chemotherapeutic agents reported that the mean inhibition rates (%) for paclitaxel and cisplatin were 46% and 54%, respectively [52]. So, the chemosensitivity assay in hydrogels might primarily predict precise drug response in vitro against clinically-therapeutic agents.

## Conclusions

To sum up, 3D cell culture in RADA16-I hydrogel was one reliable, high-profile, and proactive cell culture platform compared to the monolayer cell culture and other 3D cell culture counterparts in Matrigel or collagen I. In viable cell proliferation and active cell growth in 3D context, similar to 3D-cultured cells in Matrigel and collagen I, HO-8910PM cells in RADA16-I hydrogel showed diverse cell shapes in morphology and a variety of cell aggregate growth patterns for a long cell culture period. RADA16-I hydrogel maintained enough cell viability and high vitality of HO-8910PM cells and proper cell-nanofiber scaffold microenvironments by various cell aggregate growth patterns, such as cell aggregates, cell colonies, cell clusters, and MCTS et al. 3D-cultured HO-8910PM cells in the MCTS expressed different cell adhesion proteins, such as integrin β1, E-cadherin, and N-cadherin, which indicated the cell–cell adhesion or intercellular junction and proper cell-ECM interactions in RADA16-I hydrogel, Matrigel and collagen I. The MTT end point assay in hydrogels was developed to indicate chemosensitivity assay for clinical drug responses (paclitaxel and cisplatin) when compared with 2D flat cell culture, which represented one activatable or precise cell models to develop a high-profile 3D cell culture technique. Our results provided the basic evidences that RADA16-I hydrogel was a versatile nanofiber hydrogel scaffold to form biomimetic 3D cell culture models by bioengineering nanotechnology. Due to the versatile and bioengineering merits, designer peptide hydrogels might cater for the future clinical medicine to a high translational possibility and extensive biomedical significance in cell techniques.

## Data Availability

All data generated and analyzed during this research are included in this published article.
